# Molecular Cloning and Characterization of a *meta*/*para*-*O*-Methyltransferase from *Lycoris aurea*

**DOI:** 10.3390/ijms19071911

**Published:** 2018-06-29

**Authors:** Bin Sun, Peng Wang, Ren Wang, Yikui Li, Sheng Xu

**Affiliations:** 1 Institute of Botany, Jiangsu Province and Chinese Academy of Sciences, Nanjing 210014, China; sunbin9825@163.com (B.S.); wp437731866@163.com (P.W.); jswangren@aliyun.com (R.W.); 2 The Jiangsu Provincial Platform for Conservation and Utilization of Agricultural Gerplasm, Nanjing 210014, China

**Keywords:** *Lycoris aurea*, *O*-methyltransferase, 3,4-dihydroxybenzaldehyde, caffeic acid, norbelladine, Amaryllidaceae alkaloids

## Abstract

*O*-methyltransferases (OMTs) have been demonstrated to play key roles in the biosynthesis of plant secondary metabolites, such as alkaloids, isoprenoids, and phenolic compounds. Here, we isolated and characterized an *OMT* gene from *Lycoris aurea* (namely *LaOMT1*), based on our previous transcriptome sequencing data. Sequence alignment and phylogenetic analysis showed that LaOMT1 belongs to the class I OMT, and shares high identity to other known plant OMTs. Also, LaOMT1 is highly identical in its amino acid sequence to NpN4OMT, a norbelladine 4′-OMT from *Narcissus* sp. *aff. pseudonarcissus* involved in the biosynthesis of Amaryllidaceae alkaloids. Biochemical analysis indicated that the recombinant LaOMT1 displayed both *para* and *meta* *O*-methylation activities with caffeic acid and 3,4-dihydroxybenzaldehyde, and showed a strong preference for the *meta* position. Besides, LaOMT1 also catalyzes the *O*-methylation of norbelladine to form 4′-*O*-methylnorbelladine, which has been demonstrated to be a universal precursor of all the primary Amaryllidaceae alkaloid skeletons. The results from quantitative real-time PCR assay indicated that *LaOMT1* was ubiquitously expressed in different tissues of *L. aurea*, and its highest expression level was observed in the ovary. Meanwhile, the largest concentration of lycorine and galanthamine were found in the ovary, whereas the highest level of narciclasine was observed in the bulb. In addition, sodium chloride (NaCl), cold, polyethylene glycol (PEG), sodium nitroprusside (SNP), and methyl jasmonate (MeJA) treatments could significantly increase *LaOMT1* transcripts, while abscisic acid (ABA) treatment dramatically decreased the expression level of *LaOMT1*. Subcellular localization showed that LaOMT1 is mainly localized in cytoplasm and endosome. Our results in this study indicate that LaOMT1 may play a multifunctional role, and lay the foundation for Amaryllidaceae alkaloid biosynthesis in *L. aurea*.

## 1. Introduction

Methylation performed by *S*-adenosyl-l-methionine (SAM, AdoMet)-dependent *O*-methyltransferases (OMTs) is a common ubiquitous reaction that takes place in various organisms. OMTs catalyze the transfer of a methyl group from SAM to the hydroxyl group of an acceptor compound with the formation of its methyl ether derivatives and *S*-adenosyl-l-homocysteine (SAH) [1]. They are abundant in the biosynthetic pathways of a diverse range of natural products [2]. In plants, OMTs often contributes to the biosynthesis of compounds related to flower scents, pigments, or phytoalexins that play important roles in biological interaction [3]. OMTs also function in methylation of plant secondary metabolites including alkaloids, isoprenoids and phenolic compounds [4]. Hence, *O*-methylation is influencing a variety of processes such as plant growth, development, signaling, stress tolerance, and disease resistance [1].

In plants, OMTs can be grouped in two main classes based on amino acid sequence alignments, size, and substrate variance [4,5,6]. Class I OMTs have lower molecular weights (approximately 23–27 kDa) and require divalent cations. Most class I OMTs have been shown to be specific for caffeoyl coenzyme A esters of phenylpropanoids (CCoAOMTs), which are thought to be key enzymes in the biosynthesis of lignin [7,8]. However, subgroups of the CCoAOMTs also methylate a variety of metabolites with catechol-type functionality, including flavonoids, anthocyanins, coumarins, and aromatic esters [9,10,11,12,13]. In addition, CCoAOMTs from plants exhibit a high sequence similarity to mammalian and bacterial catechol OMTs [5,14]. With only a few exceptions, the position specificity of CCoAOMTs is always conserved and restricted towards methylation of the *meta*-hydroxyl group of catechols consistent with the vanilloid types of substitution patterns observed in plants [6,15,16,17]. Class II OMTs are larger (38–43 kDa) and cation independent, while they usually accept a broad range of substrates and utilize a histidine (His)-based catalytic dyad to facilitate their methyltransferase activities [18]. The most widely studied Class II OMTs are caffeic acid OMTs, which are involved in the synthesis of S-lignin. Although still referred to as caffeic acid OMTs, the preferred substrates are actually caffeoyl aldehyde and 5-hydroxyconiferaldehyde [19,20,21,22,23]. Further, class II OMTs also catalyze the methylation of a diverse range of secondary metabolites including flavonoids, flavonols, stilbenes, phenylpropenes, and various volatile phenolics [24,25,26,27,28]. On the other hand, the *para*-*O*-methylation activity is mostly observed from plant class II OMTs [29,30].

*Lycoris aurea* (L’ Her.) Herb, also called Golden Magic Lily, is an ornamentally and medicinally important perennial herbaceous plant belonging to the Amaryllidaceae family. It has been used in traditional Chinese medicine (TCM) for a long time. Phytochemical analysis showed that *L. aurea* contained various Amaryllidaceae alkaloids such as galanthamine, haemanthamine, and lycorine, which have been reported to exhibit medicinal values [31,32,33,34,35]. In general, Amaryllidaceae alkaloids are derived from the aromatic amino acids phenylalanine and tyrosine, which are used to produce the common precursor 4′-*O*-methylnorbelladine. And, 4′-*O*-methylnorbelladine is speculated to be the methylation product of norbelladine catalyzed by OMT [36,37,38]. Subsequently, the cyclisation of 4′-*O*-methylnorbelladine by oxidative C–C phenol coupling, which can occur in *ortho-para*’, *para-para*’ and *para-ortho*’ positions, leads to the biosynthesis of Amaryllidaceae alkaloids with different core skeletons [36,39,40]. Recently, based on the *de novo* transcriptome sequencing created for *Narcissus* sp. *aff. pseudonarcissus*, a class I *O*-methyltransferase N4OMT (NpN4OMT), which is responsible for the methylation of norbelladine to 4′-*O*-methylnorbelladine in the proposed galanthamine biosynthetic pathway was identified and characterized [38]. However, there is no information that described any homologue to NpN4OMT or other known OMTs, nor about those functions involved in the biosynthesis of Amaryllidaceae alkaloids in *L. aurea*.

In this study, an *OMT* gene, *LaOMT1*, was identified based on the previous transcriptome sequencing data of *L. aurea* [41,42]. LaOMT1 belongs to class I OMT, and is most closely related to NpN4OMT (>90% amino acids sequence identity). We found that LaOMT1 was able to display *O*-methylation activities with different substrates, including caffeic acid, 3,4-dihydroxybenzaldehyde, and norbelladine, and had a slightly different substrate range than NpN4OMT1. *LaOMT1* gene was ubiquitously expressed in different tissues of *L. aurea*, with the highest expression level in the ovary. At the same time, the largest concentration of lycorine and galanthamine were also found in the ovary, whereas the highest level of narciclasine was observed in the bulb. In addition, *LaOMT1* gene was induced by the treatments with sodium chloride (NaCl), cold, and polyethylene glycol (PEG) stresses, and sodium nitroprusside (SNP) as well as methyl jasmonate (MeJA). In contrast, the expression level of *LaOMT1* was significantly inhibited by abscisic acid (ABA) treatment. Moreover, our data from the transformed Arabidopsis protoplast and tobacco epidermal cell assays agree with that LaOMT1 could be mainly localized in cytoplasm and endosome. Taken together, our investigation on LaOMT1 may lay a foundation of research for the Amaryllidaceae alkaloid biosynthesis in *L. aurea*.

## 2. Results

### 2.1. Isolation and Characterization of LaOMT1

**Figure 1:**
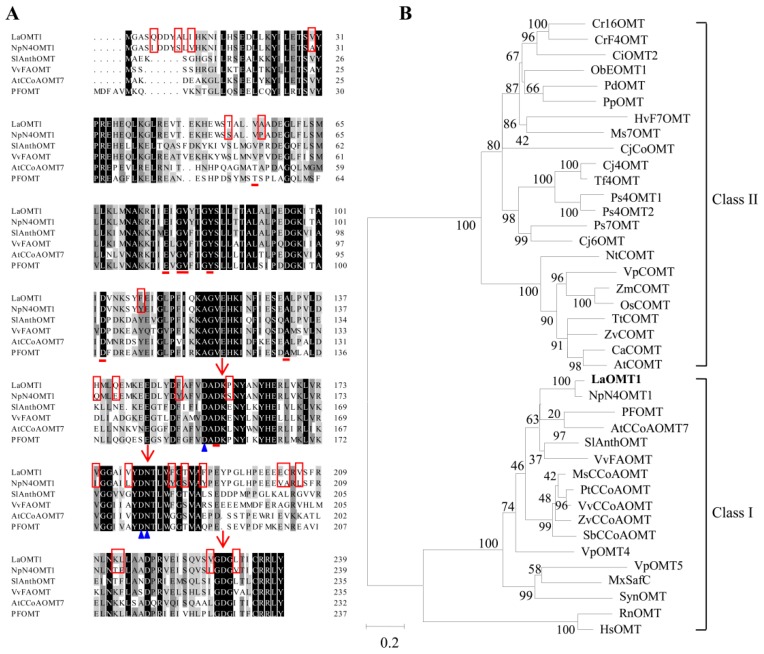
Sequence analysis of LaOMT1. (**A**) Protein sequence alignment of LaOMT1 with homologous proteins from other species. Amino acids shaded in black are identical, those in dark and light grey are similar. Amino acids involved in interactions with a methyl group donor *S*-adenosyl-l-methionine (SAM) are marked with red lines. Amino acids involved in interactions with metal ions are marked with blue triangle, and amino acids involved in the catalysis of the methyl transfer are marked with red arrows. The different amino acid residues between LaOMT1 and NpN4OMT1 are indicated as red frames. (**B**) Phylogenetic analysis of LaOMT1 and other OMTs. Alignment of sequence was performed with Clustal Omega, and the phylogenetic tree was constructed with MEGA version 5.2 (http://www.megasoftware.net) using the maximum-likelihood method with 1000 replicate bootstrap support. The characterized OMTs from different species were listed in Table S2. Numbers at the nodes indicate the percent bootstrap values. The bar at bottom shows 0.2 amino acid substitution. The LaOMT1 is highlighted in bold.

The full-length cDNA of *LaOMT1* gene was previously obtained from transcriptome sequencing of *L. aurea* [41,42]. The open reading frame (ORF) of the *LaOMT1* gene was 720 bp, encoding a 239-amino acid protein (Figure S1). And protein analysis indicated LaOMT1 had a predicted molecular weight (MW) of 26.98 kDa and pI of 5.00. Multiple amino acid sequence alignments of LaOMT1 and other characterized plant OMTs in NCBI GenBank database were performed. As shown in Figure 1A, LaOMT1 shared 90%, 64%, 63%, 56%, and 55% sequence identity with OMT proteins of *Narcissus sp*. *aff. Pseudonarcissus* (AIL54541.1), *Vitis vinifera* (C7AE94.1), *Solanum lycopersicum* (NP_001289828.1), *Arabidopsis thaliana* (NP_567739.1), and *Vanilla planifolia* (ADZ76153.1), respectively. To further investigate the evolutionary relationships among LaOMT1 and other OMTs from different species, a phylogenetic tree was constructed on the basis of the sequence similarities. Therefore, it was believed that LaOMT1 belongs to the class I OMT group (Figure 1B and Table S2).

### 2.2. Biochemical Characterization of the Recombinant LaOMT1 Protein

To verify the catalytic activity of LaOMT1, the complete coding sequences of LaOMT1 was cloned into the expression vector pET-28(a). The recombinant LaOMT1 protein containing hexa histidine (His) tag at the N-terminus was expressed in a heterologous expression system, *E. coli* BL21 (DE3). The SDS-PAGE analysis confirmed the successful expression of the LaOMT1 fusion protein with an expected MW, following induction with 1 mM isopropyl-β-d-thiogalactopyranoside (IPTG) for 16 h at 28 °C (Figure S2). Subsequently, the recombinant protein was purified from the *E. coli* lysate using Ni-affinity column chromatography (Figure S2).

**Figure 2:**
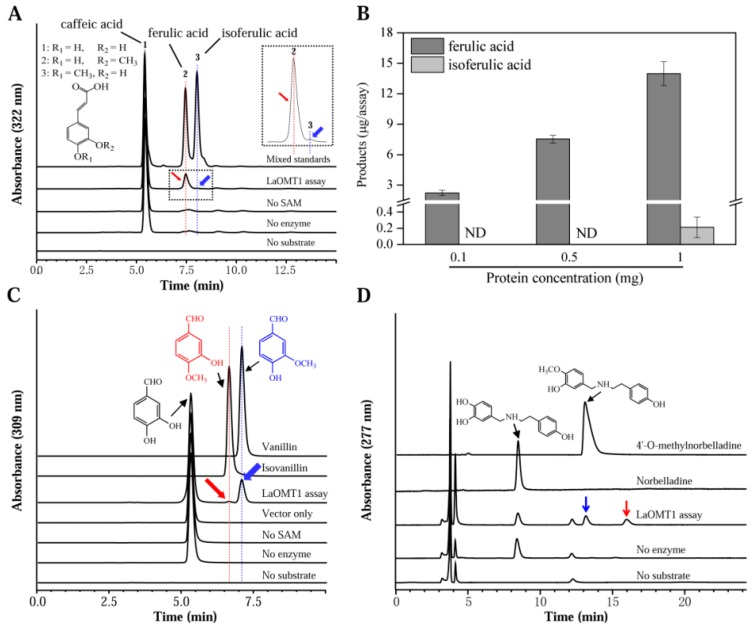
Assays of LaOMT1 enzyme activity in vitro for different substrates. (**A**) HPLC analysis of cafferic acid (1) incubated with the recombinant LaOMT1 protein. Two new peaks representing ferulic acid (2) and isoferulic acid (3) were verified, according to each identical retention time. (**B**) A dose-dependent activity of LaOMT1 when 1 mM caffeic acid incubated with the recombinant LaOMT1 enzyme in different concentrations. Each dataset represents the mean ± standard deviation (SD) from triplicate measurements. (**C**) HPLC analysis of 3,4-dihydroxybenzaldehyde incubated with the recombinant LaOMT1 protein, where two new peaks with the same retention time as isovanillin (red arrow) and vanillin (blue arrow) were found. (**D**) HPLC analysis of norbelladine incubated with the recombinant LaOMT1 protein. The two new products were indicated with blue arrow and red arrow, respectively.

To determine the substrate specificity of LaOMT1, several similar substrates were tested (Table 1). The reaction activity of LaOMT1 was first determined by measuring the catalyzation of caffeic acid. The enzyme assays with LaOMT1 yielded, upon HPLC analysis, a peak with the retention time of ferulic acid, and another peak with the retention time of isoferulic acid (Figure 2A). The absence of product in the assay lacking SAM shows that the LaOMT1 uses SAM as a co-substrate and cannot form product without SAM. Furthermore, the two new product peaks had the same mass spectrometry (MS) pattern as the ferulic acid and isoferulic acid standards, respectively (Figure S3A). Meanwhile, the effects of protein concentration on LaOMT1 activity for caffeic acid are shown in Figure 2B. Kinetic parameters of LaOMT1 for caffeic acid were also determined under initial rate conditions. LaOMT1 converted caffeic acid with an apparent *K*_m_ value of 20.32 μM and apparent *V*_max_ value of 12.90 pkat mg^−1^ protein (Figure S4).

**Figure 3:**
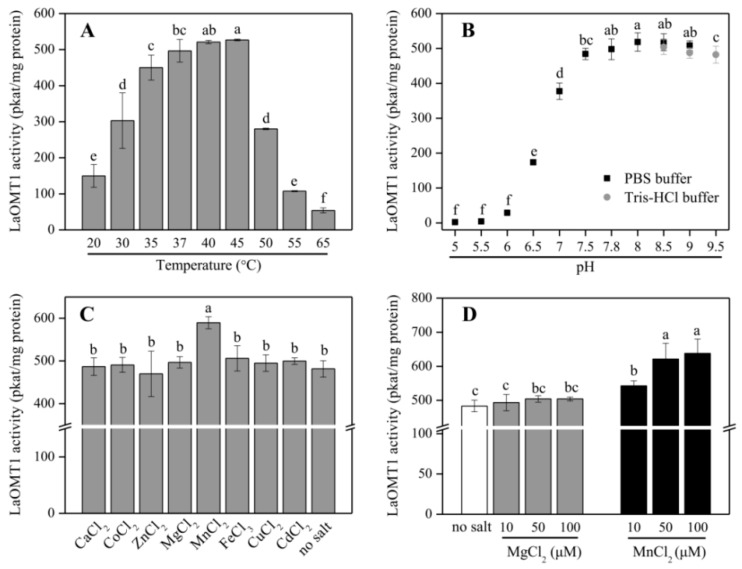
Effect of temperature (**A**), pH (**B**), divalent cations (**C**), and different concentrations of MgCl_2_ and MnCl_2_ (**D**) on LaOMT1 enzyme activity for 3,4-dihydroxybenzaldehyde. The tests contained 1 mM 3,4-dihydroxybenzaldehyde in the assay mixture.

Also, two methylated derivatives from the 3,4-dihydroxybenzaldehyde were observed, when monitoring recombinant LaOMT1 activity (Figure 2C). Actually, these two products are identical to vanillin and isovanillin, respectively (Figure 2C and Figure S3B). LaOMT1 converted 3,4-dihydroxybenzaldehyde with an apparent *K*_m_ value of 151.94 μM and apparent *V*_max_ value of 586.57 pkat mg^−1^ protein (Figure S5). The *k*_cat_ of 3,4-dihydroxybenzaldehyde was apparently higher than that observed for caffeic acid (Table 1). Moreover, by using norbelladine as the substrate, the enzyme assays of LaOMT1 also yielded two product peaks (Figure 3D). One product peak had both the same retention time and MS pattern as the 4′-*O*-methylnorbelladine. The other one only had the same MS pattern as the 4′-*O*-methylnorbelladine, which is speculated to be 3′-*O*-methylnorbelladine (Figure S3C). Additionally, under the assay conditions used, no product was yielded when vanillin, isovanillin, *O*-vanillin and tyramine were applied as the substrates, respectively (Table 1).

Further, the purified recombinant LaOMT1 protein was applied to analyze its optimal temperature (Figure 3A) and pH (Figure 3B), when incubated with 3,4-dihydroxybenzaldehyde. LaOMT1 activity increased with rising temperature within 20–45 °C, whereas, declined thereafter. The optimum pH of LaOMT1 was found to be 8.0. In addition, when testing LaOMT1 affected by cation dependence, LaOMT1 activity can be improved upon the presence of manganese in a dose-dependent pattern (Figure 3C,D).

### 2.3. Expression Analysis of LaOMT1 and Accumulation of Amaryllidaceae Alkaloids in Different Tissues

**Figure 4:**
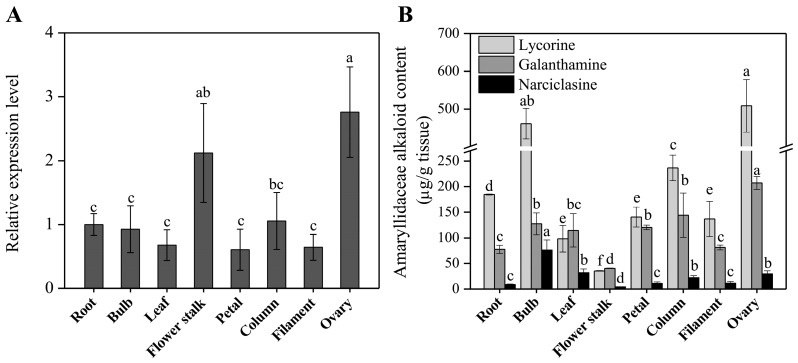
Expression level of the *LaOMT1* gene (**A**) and accumulation level of three Amaryllidaceae alkaloids (**B**) in different tissues of *L. aurea*. The expression level of *LaOMT1* was measured using qRT-PCR analysis, and normalized to the reference gene *LaEXP1*. The expression of *LaOMT1* in root tissue was defined as 1.0. Data represent the mean ± standard deviation (SD) of three independent biological replicates. Different letters indicate significant differences (*p* < 0.05) according Duncan’s multiple test.

The expression profiles of the *LaOMT1* in different tissues including root, bulb, leaf, flower stalk, and different parts of flowers (petal, column, filament and ovary) were evaluated by using quantitative real-time PCR (qRT-PCR) analysis. The results showed that *LaOMT1* ubiquitously expressed in all tissues detected, with the highest expression level in the ovary (Figure 4A). Meanwhile, the contents of three Amaryllidaceae alkaloids including lycorine, glanthamine, and narciclasine were measured. The results showed that the largest concentration of lycorine was found in ovary tissue, whereas, the lowest level in the flower stalk. Similar result was observed for the determination of galanthamine. However, the highest concentration of narciclasine was found in the bulb, with the lowest level in flower stalk (Figure 4B).

**Figure 5:**
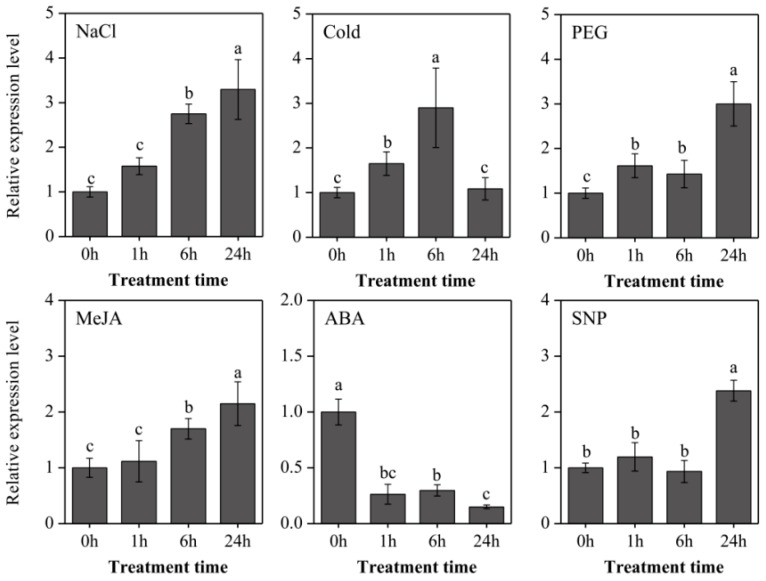
Expression patterns of *LaOMT1* during *L. aurea* roots treated with NaCl, cold, PEG, MeJA, ABA, and SNP. The expression level of *LaOMT1* was measured using qRT-PCR analysis, and normalized to the reference gene *LaTIP41*. The expression of *LaOMT1* in control sample (without treatment at 0 h) was defined as 1.0. Values were shown as means ± standard deviation (SD) of three independent biological replicates. Different letters above the bars indicate that the means are statistically different according to Duncan’s multiple test (*p* < 0.05). NaCl, sodium chloridel; PEG, polyethylene glycol; MeJA, methyl jasmonate; ABA, abscisic acid; SNP, sodium nitroprusside.

To further explore the expressional characters of *LaOMT1*, roots of young *L. aurea* seedlings were treated with three stresses (NaCl, sodium chloride for salinity stress; 4 °C for cold stress; PEG, polyethylene glycol for osmotic stress), two hormones (ABA, abscisic acid; MeJA, methyl jasmonate), and a nitric oxide (NO) donor (SNP, sodium nitroprusside). As shown in Figure 5, the *LaOMT1* expression was significantly induced by salinity stress treatment, and reached the maximum at 24 h with a 3.29-fold increase. Similar induction expression tendency of *LaOMT1* was observed under MeJA treatment. For cold stress treatment, the *LaOMT1* expression was gradually enhanced and reached a peak at 6 h with a 2.90-fold increment. However, the expression level of *LaOMT1* was significantly depressed under ABA treatment. For PEG treatment, the *LaOMT1* expression was also induced and peaked at 24 h with a 3.47-fold increase, while the induction of *LaOMT1* expression in response to SNP treatment was only observed at 24 h.

### 2.4. Subcellular Localization of the LaOMT1 Protein

**Figure 6:**
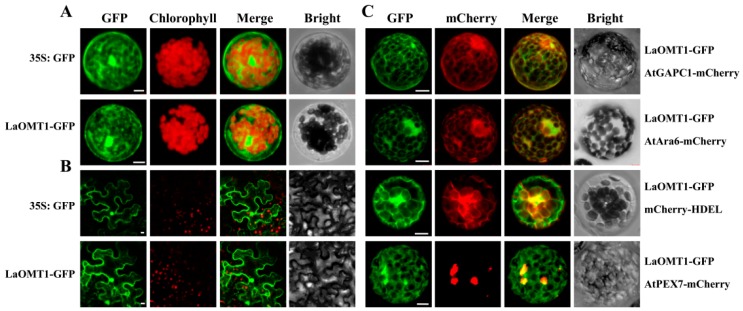
Subcellular localization of LaOMT1. (**A**) Subcellular localization of GFP alone and LaOMT1-GFP fusion protein in Arabidopsis protoplasts. (**B**) Subcellular localization of GFP alone and LaOMT1-GFP fusion protein in tobacco epidermal cells. (**C**) Protoplasts co-expressing LaOMT1-GFP and four different Arabidopsis marker proteins, i.e., a cytoplasm marker (AtGAPC1-mcherry), an endosome marker (AtAra6-mcherry), an endoplasmic reticulum (mcherry-HDEL) and a peroxisome marker (AtPEX7-mcherry), respectively. The photographs were taken in the green channel (GFP fluorescence), red channel (mCherry fluorescence), combination of green and red channel, and bright channel. Scale bar = 10 μm.

To determine the subcellular localization of the LaOMT1 protein, we transiently expressed LaOMT1 in both Arabidopsis protoplasts and tobacco epidermal cells. The LaOMT1 was fused with green fluorescent protein (GFP) as a fluorescent marker, under the control of CaMV 35S promoter. As shown in Figure 6, LaOMT1-GFP was observed in a cytosolic fluorescence pattern, very similar to that obtained with non-targeted GFP in both Arabidopsis protoplasts (Figure 6A) and tobacco epidermal cells (Figure 6B). Besides, the fluorescent marker proteins characteristic for the cytoplasm (AtGAPC1-mcherry, At3g04120), the endosome (AtAra6-mcherry, At3g54840), the endoplasmic reticulum (ER; mcherry-HDEL) [43], and the peroxisome (AtPEX7-mcherry, At1g29260) were co-expressed with LaOMT1-GFP, respectively. It showed that signals of LaOMT1-GFP were almost identical to that of AtGAPC1-mcherry (cytoplasm) or AtAra6-mcherry (endosome). Meanwhile, there were partial and/or only small proportion of LaOMT1-GFP overlapped with the red fluorescent signals of mcherry-HDEL (ER) and AtPEX7-mcherry (peroxisome). Therefore, LaOMT1 could be mainly localized in cytoplasm and endomsome.

## 3. Discussion

According to the transcriptome sequencing data of *L. aurea* [20,21], an *OMT* gene named as *LaOMT1* was identified. The amino acids sequence length of LaOMT1 is consistent with the amino acid range found in class I OMTs [4,5,6]. Additionally, alignments with other plant OMTs showed that LaOMT1 is most closely related to the class I OMT NpN4OMT (Figure 1A), and phylogenetic analysis also showed that LaOMT1 was placed in the class I OMT group (Figure 1B).

LaOMT1 was capable of methylating caffeic acid in vitro to form two products ferulic acid and isoferulic acid, and showed a strong preference for the 3-OH (*meta*) position (Figure 2A). Repeatably, similar results were observed when 3,4-dihydroxybenzaldehyde was applied as a substrate (Figure 2B). These results are consistent with the findings from mammalian and bacterial catechol OMTs, which catalyse the methylation in both *meta* and *para* position of the catechols with a *meta* preference for most substrates tested [44]. In plants, some caffeic acid OMTs have been reported to have activity against 3,4-dihydroxybenzaldehyde. For example, caffeic acid OMTs from basil [11] and strawberry [45] have activity 69.4% and 140% respectively, of catalyzing activities with 3,4-dihydroxybenzaldehyde, comparing to the relative activity with caffeic acid. Moreover, in *Vanilla planifolia*, a multifunctional OMT was isolated and demonstrated to have a broad range of substrate specificity, and this enzyme also catalyze the conversion of 3,4-dihydroxybenzaldehyde to vanillin [46]. Nevertheless, above OMTs can effectively catalyze the SAM-dependent methylation of 3,4-dihydroxybenzaldehyde and caffeic acid at the 3-OH (*meta*) position, but not at the 4-OH (*para*) position. In addition, when norbelladine was applied as a substrate, two new products were observed in the presence of LaOMT1 (Figure 2C). This result is different from that of NpN4OMT, which methylates norbelladine to form only one product, 4′-*O*-methylnorbelladine [38]. Even more, although a high sequence identity (>90%) was observed between LaOMT1 and NpN4OMT1 (Figure 1A), the substrate specificity and regioselectivity of LaOMT1 is significantly different from that of NpN4OMT, whose enzyme activity was not detectable with 3,4-dihydroxybenzaldehyde or caffeic acid as a substrate [38]. Meanwhile, sequence alignments of different plant OMTs including LaOMT1 and NpN4OMT1 showed that the amino acid residues have been identified as putatively important for SAM binding, metal ion interaction, and the catalysis of methyl transfer are conserved (Figure 1A). Previous study has showed whether catechol 4′-OMT or catechol 3′-OMT can be determined by the enzymes varying as little as one amino acid [16]. Thus, the different amino acids between NpN4OMT1 and LaOMT1 need be further identified in terms of substrate specificity. Accordingly, other factors might influence the regioselectivity of LaOMT1 should also be investigated.

The maximum enzymatic activity of LaOMT1 for 3,4-dihydroxybenzaldehyde appeared at pH 8.0, and was not significantly affected in pH buffers ranging from 8.0 to 9.5, indicating that LaOMT1 may have a certain degree of alkali resistance (Figure 3). When testing LaOMT1 for cation dependence, its enzymatic activity was improved upon the addition of manganese. This result is consistent with the conclusion that class I OMTs require divalent cations [5,18]. Hence, in terms of the wide usage of vanillin in food, medicines and cosmetics as a flavoring agent, LaOMT1 may be useful in engineering strategies for the synthesis of natural vanillin.

**Figure 7:**
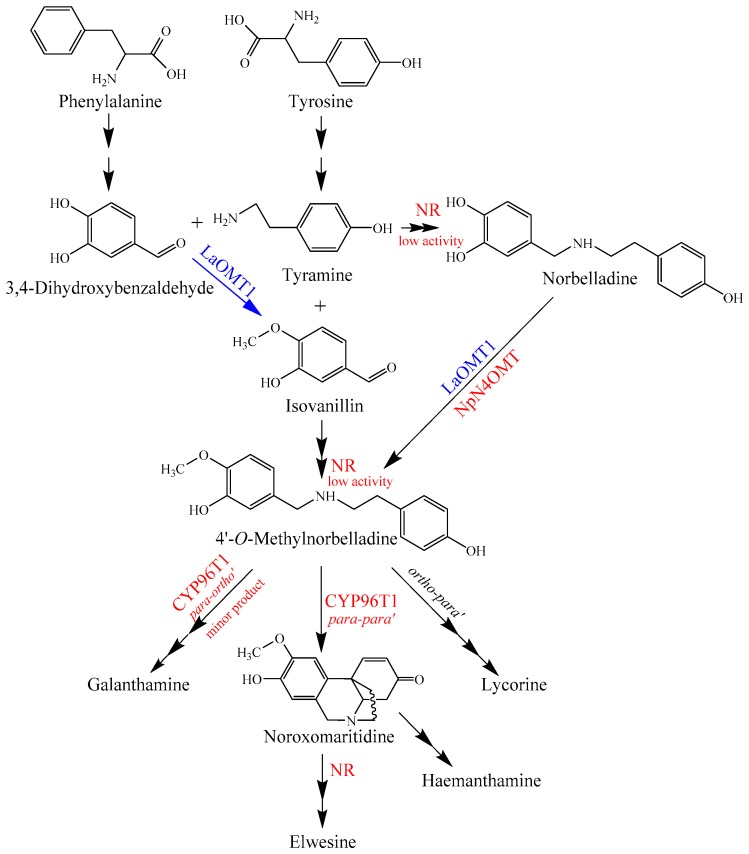
Proposed Amaryllidaceae alkaloids biosynthesis pathway in *L. aurea*. LaOMT1 involved in the pathway is indicated in blue. NpN4OMT and the possible homologues of *Narcissus* sp. *aff. Pseudonarcissus* noroxomaritidine reductase (NR) and *C-C* phenol coupling cytochrome P450 (CYP96T1) were indicated in red.

It has been established that the core biosynthetic pathway of the Amaryllidaceae alkaloids consists of the reactions required to produce 3,4-dihydroxybenzaldehyde and tyramine, the condensation and reduction of these precursors to norbelladine, and the subsequent methylation of norbelladine to 4′-*O*-methylnorbelladine [38,39]. The identification of NpN4OMT showed that it is responsible for the methylation of norbelladine to 4′-*O*-methylnorbelladine in Amaryllidaceae alkaloid biosynthesis [38]. In this study, LaOMT1 could catalyze the methylation of norbelladine to form 4′-*O*-methylnorbelladine, as well as the formation of isovanillin from 3,4-dihydroxybenzaldehyde, suggesting that methylation might occur prior to formation of norbelladine in *L. aurea* (Figure 7). Interestingly, a noroxomaritidine reductase (NR), which co-expresses with the NpN4OMT, was identified from *N.* sp. *aff. Pseudonarcissus*, recently [40]. Although conducting much lower than noroxomaritidine conversion, the NR with a specific activity is capable to produce norbelladine from 3,4-dihydroxybenzaldehyde and tyramine and to produce 4′-*O*-methylnorbelladine from isovanillin and tyramine as well [40]. This substrate flexibility of NR further indicated that the diverse sources for forming 4′-*O*-methylnorbelladine within the Amaryllidaceae (Figure 7). Thus, comparing to previous works on NpN4OMT [38] and CYP96T1 [39], to identify NR together with LaOMT1 may also provide biochemical insight on Amaryllidaceae alkaloid biosynthesis in *L. aurea* (Figure 7). Certainly, role of such specified enzymes in the proposed Amaryllidaceae alkaloids biosynthetic pathway in *L. aurea* should be further investigated.

Genes involved in the biosynthetic pathway of plant metabolites are often co-regulated and this leads to correlations between biosynthetic gene expression and corresponding end product accumulation [47,48,49]. In *N.* sp. *aff. pseudonarcissus*, the discovery of the Amaryllidaceae alkaloid biosynthetic genes *NpN4OMT* [38], *C-C* phenol coupling cytochrome P450 (*CYP96T1*) [39], and *NR* [40] through correlations with galanthamine accumulation (*N4OMT*) and co-expression with *N4OMT* (CYP96T1 and *NR*) were also observed. In this study, the highest expression level of *LaOMT1* and the largest concentration of lycorine and galanthamine were observed in the ovary (Figure 2), suggesting a correlation between Amaryllidaceae alkaloids accumulation and *LaOMT1* gene expression in this tissue. However, the inconsistency between Amaryllidaceae alkaloids accumulation and *LaOMT1* gene expression was also observed in bulb and flower stalk tissue. This might be caused by the different time in development between these two tissues.

The production of Amaryllidaceae alkaloids was strongly influenced by chemical factors, such as plant growth regulators, sucrose, macronutrients, and elicitation, as well as physical factors including temperature, light, and physical state of the medium [50,51]. For example, the auxins, cytokinins, and ethylene on the capacity of tissue cultures for Amaryllidaceae alkaloid accumulation were observed [52,53,54]. The most common elicitor methyl jasmonate (MeJA) could significantly promote Amaryllidaceae alkaloids biosynthesis in Amaryllidaceae [55,56]. High sucrose levels by changing the osmotic pressure resulted in the promoted production of galanthamine by shoot cultures [57,58]. The nitric oxide (NO) dornor, sodium nitroprusside (SNP) also enhanced the contents of galanthamine in genus *Lycoris* [59,60]. In addition, precursors of the Amaryllidaceae alkaloid biosynthesis pathway, such as 4′-*O*-methylnorbelladine, also affected the contents of Amaryllidaceae alkaloids [61,62]. In this study, the experiments were performed to investigate the expressional characters of *LaOMT1* under different treatment condition. Consistent with previous researches on Amaryllidaceae alkaloid accumulation under SNP and MeJA treatment, the inducible expression patterns of *LaOMT1* were also detected (Figure 3). In addition, our results also showed that the *LaOMT1* expression was significantly induced by NaCl, PEG, and cold stress treatment, whereas inhibited by ABA treatment. Still, the detailed influences of these treatments on Amaryllidaceae alkaloids should be further illuminated.

Most of the plants OMTs have been reported as cytosolic proteins in soluble form [10,63,64,65]. In this study, the LaOMT1-GFP fusion protein was presented in a cytosolic fluorescence pattern (Figure 6A,B), almost identical to the red fluorescence of AtGAPC1-mcherry (Figure 6C), which is regarded as a cytosolic protein [66]. Meanwhile, the subcellular colocalization of the LaOMT1-GFP performed with AtAra6-mcherry indicated their presence in endosomes (Figure 6C). Also, a portion of the LaOMT1-GFP overlapped with mcherry-HDEL, suggesting the potential localization of LaOMT1 in ER. In consist, the ER localization of alfalfa isoflavone *O*-methyltransferase (IOMT) was also observed previously [67]. Thus, our results support a notion that LaOMT1 could be mainly localize into cytoplasm and endosome. The subcellular localization of LaOMT1 might be correlated with the substrate specificity of LaOMT1. For example, caffeic acid was found only in the cytoplasm of carrot roots and was present in the conjugated form [68]. However, the combination research of LaOMT1 localization and substrates distribution should be further investigated.

## 4. Materials and Methods

### 4.1. Plant Materials, Growth Conditions, and Treatments

The bulbs of *L. aurea* with the same or similar sizes (3.8–4.2 cm) in diameter were grown at the research station of Institute of Botany, Jiangsu Province and Chinese Academy of Sciences, Nanjing, China. *L. aurea* is a groundcover plant appearing in autumn, and its floral stems and flowers start growing from August to September, and the leaves grow from September to October. For *LaOMT1* expression analysis, different tissues of *L. aurea* including flower stalk, petal, column, filament and ovary were taken during the flowering time, whereas the samples of root, bulb, and leaf from the same plants were collected at vigorous vegetative growth stage. For salinity, drought, SNP, ABA, and MeJA treatment, *L. aurea* seedlings (with 2–3 leaves) were grown in plastic pots containing half-strength Hoagland’s nutrient solution at 22 °C under a 14/10 h day/night rhythm. After seven days maintenance, *L. aurea* seedlings were imposed in 400 mM sodium chloride (NaCl), 20% PEG-6000 solution (*w*/*v*), 0.5 mM SNP, 0.1 mM ABA, or 0.1 mM MeJA for 0, 1, 6, and 24 h respectively. For cold stress, plants were placed at chamber with the temperature of 4 °C for 0, 1, 6, and 24 h. Afterwards, the roots were harvested from three replicate plants, frozen in liquid nitrogen and stored at –80 °C [36].

### 4.2. Amaryllidaceae Alkaloids Extraction and Quantification

Different tissues of *L. aurea* were extracted by grinding with mortar and pestle cooled with liquid nitrogen, according to the method described before [69]. Three volumes of 70% ethanol were added followed by vortexing 10 min and centrifuging at 14,000× *g* for 10 min. The supernatant was filtered through a 0.22 μm sterile filter membrane. For lycorine, galanthamine and narciclasine quantitation, samples were injected (10 μL) onto an LC–20A HPLC system equipped with a SPD-M20A Photodiode Array Detector (Shimadzu Corporation, Tokyo, Japan). Separation was carried out on a reverse–phase column (InertSustain C_18_, 5 μm, 4.6 mm i.d. × 250 mm) with a flow rate of 0.8 mL/min; solvent A was 0.3% di-*n*-butylamine in H_2_O and solvent B acetonitrile. The elution system was 0–60 min, 5–50% of B, and the column was maintained at 35 °C with detection at 290 nm. Products were measured by comparing the area of the individual peaks with standard curves obtained from standard compounds.

### 4.3. Total RNA Extraction and cDNA Synthesis

Total RNA was extracted from the samples using RNAprep Pure Plant Kit (Tiangen Biotech, Beijing, China) according to the manufacturer’s instructions. The first strand cDNA was synthesized using the primescript™ RT reagent kit (TaKaRa Bio Inc., Dalian, China).

### 4.4. Cloning and Sequence Analysis of LaOMT1 Gene

The coding sequence of *LaOMT1* was amplified by PCR with specific primers (Table S1). The full-length ORF sequence of *LaOMT1* was translated using DNAMAN software. The amino acid sequences of different OMTs were aligned using Clustal Omega (http://www.clustal.org/omega/). For phylogenetic analysis, 39 OMT proteins from 28 species (Table S2) were constructed by using MEGA version 5.2 with the neighbor joining method with 1000 replicate bootstrap support.

### 4.5. Prokaryotic Expression and Purification of LaOMT1

The ORF of *LaOMT1* was amplified with a pair of specific primers (Table S1). The PCR product was digested and inserted into pET-28a (+) for heterologous expression in *Escherichia coli* strain BL21 (DE3) pLysS. When the transformed cells were incubated to an optical density at 600 nm (OD_600_) of 0.6, a final concentration of 1 mM IPTG was added to induce *LaOMT1* gene expression. The cells were harvested for SDS-PAGE analysis, and the recombinant LaOMT1 protein was purified from the *E. coli* lysate using Ni-affinity column chromatography following the manufacturer’s instructions. Protein concentrations were determined using Bradford’s assay [70].

### 4.6. Measurement of LaOMT1 Activity

The substrates norbelladine and 4′-*O*-methylnorbelladine were synthesized according to the methods reported previously [71]. The enzyme assays were performed in a final volume of 1 mL 50 mM potassium phosphate buffer (PBS, pH 7.8) containing 0.5 mM SAM, 100 µg of purified LaOMT1 protein and different concentrations of substrate (e.g., 1 mM 3,4-dihydroxybenzaldehyde, 1 mM caffeic acid, or 100 μM norbelladine). The assays were incubated for 1 h at 37 °C. Methanol (MeOH) was then added to a final concentration of 33% into the assays to quench the reactions. After filtering through 0.22-μm nylon column, the assay samples were analyzed by HPLC system. Separation was carried out on a reverse–phase column using 0.1% formic acid in water (A) versus MeOH + 0.1% formic acid (B) and run at 0.8 mL min^−1^. Products were measured by comparing the area of the individual peaks with standard curves obtained from standard compounds.

Liquid chromatography–mass spectrometry (LC–MS) was carried out on an Agilent 1260 UPLC-DAD-6530 ESI-QTOF mass spectrometer (Agilent Technologies, Santa Clara, CA, USA). Separation was on a Zorbax SB-C_18_ column (100 mm × 4.6 mm i.d., 1.8 μm) (Agilent Technologies, Santa Clara, CA, USA) using the same gradient described above. Samples were detected by positive electrospray ionization (ESI) MS using an ion source voltage of 4.0 KV and a capillary offset voltage of 80.0 V. Mass spectra were recorded from *m*/*z* 200–2000. For kinetics measurements, different substrate at varying concentrations (0.01 to 0.2 mM for caffeic acid and 0.02 to 4 mM for 3,4-dihydroxybenzaldehyde) was added to the reaction system as above. *K*_m_ and *V*_max_ values were evaluated by nonlinear regression to the Michaelis-Menten kinetics equation with the Origin 8.5 software (OriginLab Corp., Northampton, MA, USA).

### 4.7. Quantitative Real-Time PCR (qRT-PCR) Analysis

To analyze the expression levels of *LaOMT1* in different tissues or under SNP and MeJA treatment, qRT-PCR was performed using a qTOWER2.2 Real-Time Thermal Cycler (Analytik Jena AG, Jena, Germany) with One Step SYBR PrimerScript^TM^ RT-PCR Kit (TaKaRa Bio Inc., Dalian, China). The appropriate reference genes *TIP41* (*TIP41-like protein*) and *EXP2* (*Expressed protein 2*) were selected according to the previous study [69]. The relative expression levels were determined according the 2^−ΔΔ*C*t^ method. The specific primers used for qRT-PCR were listed in Table S1.

### 4.8. Subcellular Localization Analysis

For subcellular localization of LaOMT1 protein, the coding regions of *LaOMT1* was amplified by PCR using specific primers (Table S1). The PCR product was then introduced into pAN580 vector carrying a double CaMV 35S promoter for N-terminal green fluorescent protein (GFP) fusion. The well-established fluorescent protein marker mCherry-HDEL for the ER was used [43]. Other fluorescent protein markers used for the cytoplasm (AtGAPC1-mcherry, At3g04120), the endosome (AtAra6-mcherry, At3g54840), and the peroxisome (AtPEX7-mcherry, At1g29260) were also constructed in the fusion with mCherry protein of a modified vector P16ΔS:sXVE:mCherryC. The transient expression of *LaOMT1-GFP* fusion genes in Arabidopsis mesophyll protoplasts was performed following previous method [72]. In addition, the LaOMT1-GFP construct was introduced into *Agrobacterium tumefaciens* strain EHA105, which was then transformed into epidermal cells of *Nicotiana benthamiana* [73]. The transformed protoplasts and *N. benthamiana* epidermal cells were observed with a confocal laser-scanning microscope (Zeiss LSM780 META, Jena, Germany).

### 4.9. Statistical Analysis

Where indicated, results were expressed as the means ± standard deviation (SD) of three independent experiments. Statistical analysis was performed with one-way analysis of variance (ANOVA), taking *p* < 0.05 as significant according to Duncan’s multiple range test. The SPSS software version 10.0 (SPSS, Inc., Chicago, IL, USA) was used for the statistical analysis.

## Figures and Tables

**Table 1:** Substrate specificity of LaOMT1.

Substrate	Product		*K*_m_ (μM)	*K*_cat_ (min^−1^)	*K*_cat_/*K*_m_ (min^−1^ mM^−1^)
caffeic acid	ferulic acid	isoferulic acid	20.32 ± 4.81	0.023 ± 0.001	1.12
3,4-dihydroxybenzaldehyde	vanillin	isovanillin	151.94 ± 23.32	1.04 ± 0.04	6.83
norbelladine	3′-*O*-methylnorbelladine	4′-*O*-methylnorbelladine	-	-	-
vanillin	ND	ND	ND	ND	ND
isovanillin	ND	ND	ND	ND	ND
*o*-vanillin	ND	ND	ND	ND	ND
tyramine	ND	ND	ND	ND	ND

ND stands for “not detectable”. - represents product made by LaOMT1 but not quantified.

## Supplementary Materials

Supplementary materials can be found at http://www.mdpi.com/1422-0067/19/7/1911/s1.

## Abbreviations

SAM	*S*-adenosyl-l-methionine
OMT	*O*-methyltransferase
SAH	*S*-adenosyl-l-homocysteine
CCoA	caffeoyl coenzyme A
TCM	traditional Chinese medicine
PEG	polyethylene glycol
SNP	sodium nitroprusside
MeJA	methyl jasmonate
ABA	abscisic acid
MW	molecular weight
IPTG	isopropyl-thio-β-d-galactoside
GFP	green fluorescent protein
His	histidine
ORF	open reading frame
qRT-PCR	quantitative real-time PCR
LC-MS	liquid chromatography–mass spectrometry
ESI	electrospray ionization
ER	endoplasmic reticulum
ANOVA	analysis of variance
NaCl	sodium chloride
