# Supplementary material for: Molecular Cloning and Characterization of a meta/para-O-Methyltransferase from Lycoris aurea
Source: Int J Mol Sci. 2018 Jun 29;19(7):1911. doi: 10.3390/ijms19071911 (PMC6073595; doi:10.3390/ijms19071911)

Supplementary Materials:

Table S1. The list of primers used in this study.

Gene name	Species (GenBank)	Primer sequences (5'→3')	Usage
LaOMT1	*L. aurea*	P1: 5′-ATGATGGGTGCTAGCCAAGATGATTATG-3′<br>P2: 5'-TTATCAATAAAGACGTCGACAAATAGTCA-3′	*LaOMT1* ORF amplification
LaOMT1	*L. aurea*	P3: 5′-CAGCCATATGATGGGTGCTAGCCAAGATGATTATG-3′<br>P4: 5'-CGCACTCGAGTCAATAAAGACGTCGACAAATAGTCAGTC-3′	LaOMT1 protein expressions in *E. coli*
LaOMT1	*L. aurea*	P5: CAGCTCTAGAATGGGTGCTAGCCAAGATGATTATG-3′<br>P6: CGCAGGATCCATAAAGACGTCGACAAATAGTCAGTC-3′	Subcellular localization
LaOMT1	*L. aurea*	P7: 5′-CAACACGCTCTGGTTCGGAACT-3′<br>P8: 5′-CGACTGAGACTTGGGATATC-3′	qRT-PCR analysis
LaTIP41	*L. aurea*	P9: 5′-GCAACCATCCAAAGTTTAACTGCT-3′<br>P10: 5′-AATGTGCAAGCAGGGCTAGTAA-3′	Reference gene for qRT-PCR analysis
LaEXP1	*L. aurea*	P11: 5′- ATTGAAACAACCTACACCGCAA-3′<br>P12: 5′- GCTGTAAGAATGCTAATCGTTCA-3′	Reference gene for qRT-PCR analysis
AtPEX7	Arabidopsis (At1g29260)	P13: 5′-CGCTTCTAGAATGCCGGTGTTCAAAGCTCC-3′<br>P14: 5′-AATCCCCGGGACTGGCTCTAGGATCCATCCC-3′	Subcellular localization for marker protein
AtGAPC1	Arabidopsis (At3g04120)	P15: 5′-CGCTTCTAGAATGGCTGACAAGAAGATTAG-3′<br>P16: 5′-AATCCCCGGGGGCCTTTGACATGTGGACGAT-3′	Subcellular localization for marker protein
AtAra6	Arabidopsis (At3g54840)	P17: 5′-CGCTTCTAGAATGGGATGTGCTTCTTCTCTT-3′<br>P18: 5′-AATCCCCGGGTGACGAAGGAGCAGGACGAGGT-3′	Subcellular localization for marker protein

Table S2. GenBank accession numbers of OMT proteins in Fig. 1

Protein names	Species	Accession number	Class type
LaOMT	*Lycoris aurea*		I
AtCOMT	*Arabidopsis thaliana*	NP_200227.1 (At5g54160)	II
AtCCoAOMT7	*Arabidopsis thaliana*	NP_567739.1 (At4g26220)	I
Cr16OMT	*Catharanthus roseus*	ABR20103.1	II
CrF4OMT	*Catharanthus roseus*	AAR02420.1	II
CiOMT2	*Carapichea ipecacuanha*	BAI79244.1	II
CaCOMT	*Chrysosplenium americanum*	AAA86982.1	II
Cj4OMT	*Coptis japonica*	BAB08005.1	II
Cj6OMT	*Coptis japonica*	BAB08004.1	II
CjCoOMT	*Coptis japonica*	BAC22084.1	II
HsOMT	*Homo sapiens*	NP_000745.1	I
HvF7OMT	*Hordeum vulgare subsp. vulgare*	CAA54616.1	II
Ms7OMT	*Medicago sativa*	AAC49927.1	II
MsCCoAOMT	*Medicago sativa*	AAC28973.1	I
MxSafC	*Myxococcus xanthus*	AAC44130.1	I
NpN4OMT	*Narcissus* sp. *aff. Pseudonarcissus*	AIL54541.1	I
NtCOMT	*Nicotiana tabacum*	CAA50561.1	II
ObEOMT1	*Ocimum basilicum*	AAL30424.1	II
OsCOMT	*Oryza sativa*	Os08g0157500	II
Ps7OMT	*Papaver somniferum*	ACN88562.1	II
Ps4OMT1	*Papaver somniferum*	AAP45313.1	II
Ps4OMT2	*Papaver somniferum*	AAP45314.1	II
PtCCoAOMT	*Populus tremuloides*	AAA80651.1	I
PdOMT	*Prunus dulcis*	CAA11131.1	II
PfOMT	*Mesembryanthemum crystallinum*	AY14552.1	II
PpOMT	*Pyrus pyrifolia*	BAA86059.1	II
RnOMT	*Rattus norvegicus*	NP_036663.1	I
SlAnthOMT	*Solanum lycopersicum*	NP_001289828.1	I
SbCCoAOMT	*Sorghum bicolor*	XP_002436550	I
SynOMT	*Synechocystis sp. Strain PCC 6803*	WP_010873795.1	I
TtCOMT	*Thalictrum tuberosum*	AAD29842.1	II
Tf4OMT	*Thalictrum flavum subsp. glaucum*	AAU20768.1	II
VpCOMT	*Vanilla planifolia*	AAS64572.1	II
VpOMT5	*Vanilla planifolia*	ADZ76154.1	I
VpOMT4	*Vanilla planifolia*	ADZ76153.1	I
VvCCoAOMT	*Vitis vinifera*	CAA90969.1	I
VvFAOMT	*Vitis vinifera*	C7AE94.1	I
ZmCOMT	*Zea mays*	AAB03364.1	II
ZvCOMT	*Zinnia violacea*	AAA86718.1	II
ZvCCoAOMT	*Zinnia violacea*	AAA59389.1	I

Figure S1. Nucleotide and predicted protein sequence of LaOMT1.

```
1     ATGGGTGCTAGCCAAGATGATTATGCACTAATCCACAAGAATATTTTGCATAGTGAAGAT
1      M  G  A  S  Q  D  D  Y  A  L  I  H  K  N  I  L  H  S  E  D
61    CTTCTTAAGTACATATTGGAGACTAGTGTTTACCCAAGAGAGCATGAACAGCTCAAGGGG
21     L  L  K  Y  I  L  E  T  S  V  Y  P  R  E  H  E  Q  L  K  G
121   TTGAGGGAGGTGACTGAGAAACATGAATGGAGTACGGCGCTTGTCGCAGCCGATGAAGGA
41     L  R  E  V  T  E  K  H  E  W  S  T  A  L  V  A  A  D  E  G
181   TTATTTCTTTCTATGTTGTTAAAGCTCATGAATGCCAAGAGAACCATTGAGATTGGTGTA
61     L  F  L  S  M  L  L  K  L  M  N  A  K  R  T  I  E  I  G  V
241   TACACCGGTTATTCTCTGCTCACAACCGCTTTGGCTTTACCAGAAGATGGAAAGATAACG
81     Y  T  G  Y  S  L  L  T  T  A  L  A  L  P  E  D  G  K  I  T
301   GCAATTGACGTCAACAAGTCCTACTTTGAGATAGGACTGCCATTTATTCAGAAAGCAGGA
101    A  I  D  V  N  K  S  Y  F  E  I  G  L  P  F  I  Q  K  A  G
361   GTTGAGCATAAGATCAATTTCATTGAATCAGAAGCACTTCCTGTTCTTGATCATATGCTT
121    V  E  H  K  I  N  F  I  E  S  E  A  L  P  V  L  D  H  M  L
421   CAAGAGATGAAGGAAGAAGACCTCTACGACTTTGCATTTGTCGATGCAGACAAACCAAAC
141    Q  E  M  K  E  E  D  L  Y  D  F  A  F  V  D  A  D  K  P  N
481   TATGCTAATTACCACGAGCGATTAGTGAAGCTTGTCAGGGTTGGAGGAGCAATCGTCTAC
161    Y  A  N  Y  H  E  R  L  V  K  L  V  R  V  G  G  A  I  V  Y
541   GACAACACGCTCTGGTTCGGAACTGTAGCATTTCCAGAATATCCAGGCCTTCATCCAGAA
181    D  N  T  L  W  F  G  T  V  A  F  P  E  Y  P  G  L  H  P  E
601   GAGGAGGAGTGTAGGGTCTCTTTCAGAAACCTGAATAAGCTCTTGGCAGCTGATCCCCGT
201    E  E  E  C  R  V  S  F  R  N  L  N  K  L  L  A  A  D  P  R
661   GTCGAGATATCCCAAGTCTCAGTCGGCGATGGACTGACTATTTGTCGACGTCTTTATTGA
221    V  E  I  S  Q  V  S  V  G  D  G  L  T  I  C  R  R  L  Y  *
```

Figure S2. Expression and purification of N-terminal 6 × histidine-tagged LaOMT1 protein. *E. coli* BL21 (DE3) harbouring pET-28a or pET28a-LaOMT1 was incubated with or without 1 mM IPTG (isopropyl-β-d-thiogalactopyranoside) at 30 °C for 16 h. Protein samples were separated on a 10% SDS-PAGE followed by Coomassie blue staining. M, molecular mass standards; lane 1, total proteins in 20-μl aliquots from *E. coli* BL21 culture harboring pET28a; lane 2, total proteins in 20-μl aliquots from *E. coli* BL21 culture harboring pET28a induced by IPTG; lane 3, total proteins in 20-μl aliquots from *E. coli* BL21 culture harboring pET28a-LaOMT1; lane 4, total proteins in 20-μl aliquots from *E. coli* BL21 culture harboring pET28a-LaOMT1 induced by IPTG; lane 5, 10 μg recombinant LaCOMT1 protein purified by affinity chromatography. Arrow indicates the position of the fusion protein of LaOMT1.

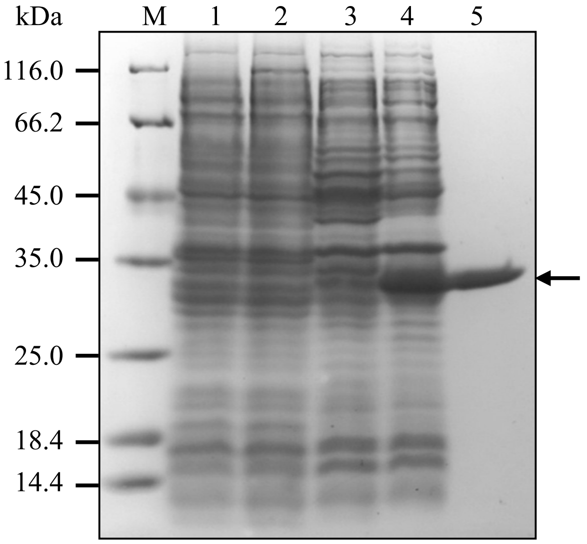

Figure S3. Mass spectrometry (MS) of new compounds produced by LaOMT1 enzyme in catalyzing the substrate caffeic acid (A), 3,4-dihydroxybenzaldehyde (B), and noberlladine (C). 1, substrate caffeic acid; 2, ferulic acid produced by LaOMT1; 3, isoferulic acid produced by LaOMT1; 4, substrate 3,4-dihydroxybenzaldehyde; 5, isovanillin produced by LaOMT1; 6, vanillin produced by LaOMT1; 7, substrate norbelladine; 8, 4'-*O*-methylnorbelladine; 9, 3'-*O*-methylnorbelladine.

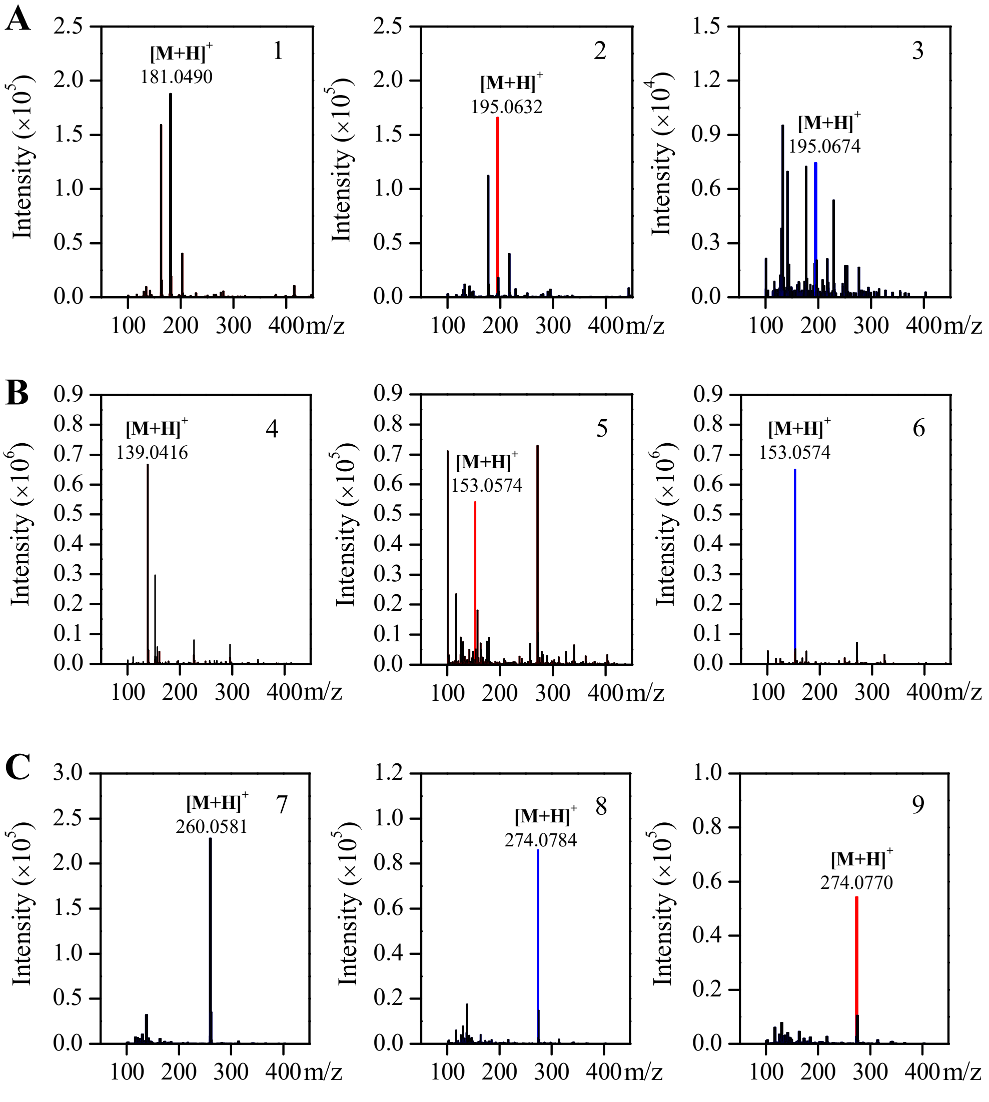

Figure S4. Measurement of the K_m and V_{max} of LaOMT1 for substrate caffeic acid.

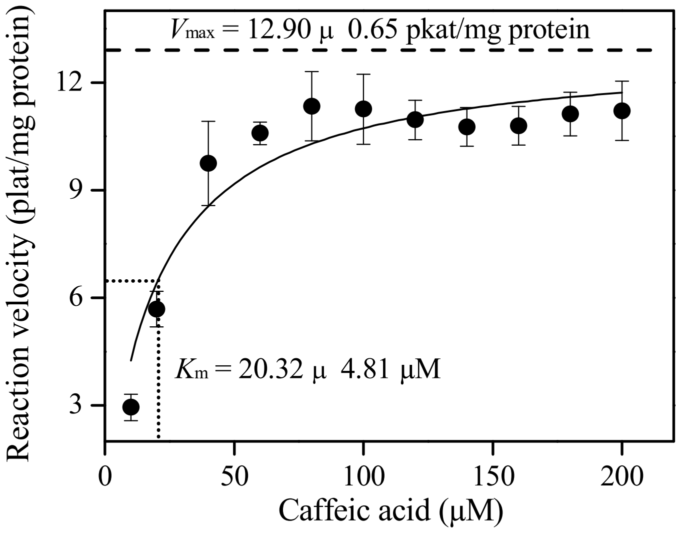

Figure S5. Measurement of the K_m and V_{max} of LaOMT1 for substrate 3,4-dihydroxybenzaldehyde.

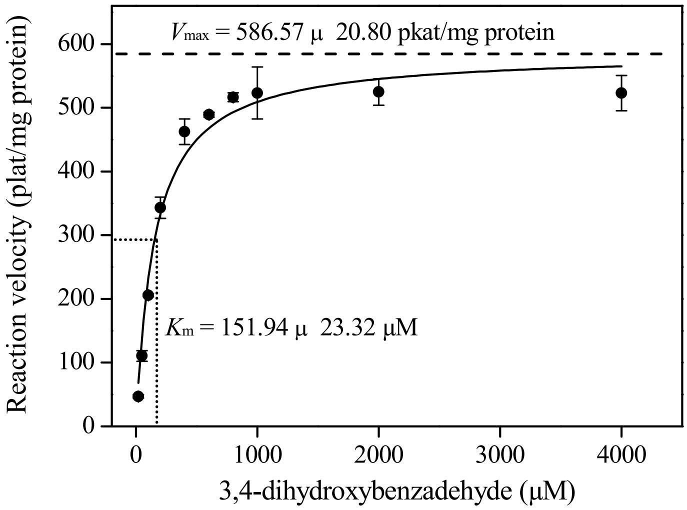

Supplement: Supplementary file 1 [file ijms-19-01911-s001.pdf]
